# Rapid spectral shaping for time domain and swept source full field OCT

**DOI:** 10.1364/BOE.574799

**Published:** 2025-10-31

**Authors:** Dimitri Roueff, Pedro Mecê, Olivier Thouvenin

**Affiliations:** Institut Langevin, ESPCI Paris, Université PSL, CNRS, 75005 Paris, France

## Abstract

Full-field optical coherence tomography (FFOCT) has recently regained attention thanks to the development of high-resolution dynamic OCT and cross-talk-free swept source FFOCT. However, the choice of wavelength and axial resolution is often a limiting factor with few existing commercial solutions. Here, we developed a novel method to provide rapid spectral shaping for FFOCT imaging. Combining a supercontinuum laser, a fast controllable acousto-optic tunable filter (AOTF), and a multimode fiber with passive and active mode mixing, we obtained an extremely flexible light source compatible with FFOCT. By tuning the AOTF frequency and integrating the resulting wavelength over one camera exposure time, it becomes possible to build any spectrum of interest in the 575-1000 nm range in time domain FFOCT. Alternatively, the designed source module enables achieving swept source FFOCT at up to 100 kfps at an unprecedented axial resolution of 1.1 *μm*.

## 1. Introduction

Full-field OCT (FFOCT) [1–3] is a variant of OCT that enables the detection of interferences in an *en face* plane conjugated to a 2D camera, without the need for transverse scanning, possibly enabling faster acquisition rates. While time domain FFOCT (TD FFOCT) is particularly useful to avoid a compromise between transverse resolution and imaging depth, enabling sub-micrometric resolution [3,4], it still requires axial scanning to achieve 3D imaging [5]. In contrast, swept-source FFOCT [6,7] (SS FFOCT) which does not require any mechanical scanner, has recently regained interest with the advent of high-speed cameras [8], and cross-talk free illumination configurations [9,10]. Indeed, a drawback of FFOCT is that its 2D detection scheme prevents the use of a spatial pinhole, which lowers the sensitivity [3,11,12] and makes FFOCT prone to cross-talk [13]. Hence, FFOCT can only reach high performances when operated with a spatially incoherent light source [9,13,14] able to form a virtual confocal pinhole [15]. Unfortunately, there is a limited number of available high-power light sources with both low spatial and temporal coherence, making it challenging to find an optimal light source for FFOCT. Recently, the development of STOC-T [9,10,16] has demonstrated the use of spatially coherent laser sources in SS- FFOCT. This was achieved by breaking the spatial coherence with passive and active mode mixing either in free-space [9] or in a multimode fiber [16,17]. However, to our knowledge, this principle has so far been validated only for commercial swept source-lasers, with infrared central wavelength and limited spectral width, limiting accessible transverse and axial resolutions in SS FFOCT, scattering strength, and making it difficult to record spectroscopic information. This is of particular importance for dynamic FFOCT [18–21], where small movements of weakly scattering organelles are analyzed [19,22].

In this article, we demonstrate the use of a novel source module for rapid spectral shaping in FFOCT. We combined a supercontinuum laser with a fast acousto-optic tunable filter (AOTF), which can switch its frequency and tune the output light central wavelength every 3 μs from a simple voltage analog signal. By sending complex voltage sequences with careful combination with the FFOCT camera exposure time, it hence becomes possible to achieve spectral shaping of the detected light. Then, by coupling this device with a carefully chosen multimode fiber with passive and active mode mixing, we can tune both the spatial and temporal coherence of the light entering a FFOCT setup, making it compatible with both TD FFOCT and SS FFOCT. For TD FFOCT, a series of wavelengths is integrated over the exposure of the detector. Each wavelength is applied for a specific illumination time, enabling the reconstruction of any complex spectrum of interest. Here, we demonstrate that such a source module can achieve high-resolution TD FFOCT and compared image quality with a traditional LED illumination. More interestingly, we demonstrate its ability to measure quantitative spectroscopic information in FFOCT by making successive measurements with variable central wavelengths, while keeping the axial resolution constant.

Alternatively, SS FFOCT was demonstrated by linearly scanning the illumination wavevector during a multi-frames acquisition. In SS FFOCT also, the total spectrum can be shaped, by modulating both the camera exposure time and the AOTF central frequency. A careful calibration of the AOTF response enables to achieve efficient linear k-sweeping. We report on ultra-high axial resolution SS FFOCT on onion layers and zebrafish larva, and show what we believe to be the highest axial resolution achieved in swept source OCT (1.0 µm full width at half maximum, compared to around 5 *μm* for high resolution swept source (FF)OCT systems [23,24]).

All in all, we developed a new source module for time domain and swept-source FFOCT, with which the spatial and temporal coherence of light can be tuned on large scales. While supercontinuum has already been reported in FFOCT [25], their spatial coherence was so far preventing their use for biological imaging. While supercontinuum coupled with AOTF has also already been demonstrated for hyperspectral imaging [26] and fluorescence microscopy [27], it was never used in OCT, especially due to the slow modulation around a few Hz, enabled by most commercial AOTFs, preventing their use for spectral shaping and fast wavelength tuning. Finally, spectral shaping has already been used in OCT [28–30], both to improve axial resolution [28], improve k linearity to reduce phase noise [29] and allow en face images reconstruction, or reduce side axial PSF sidelobes [30]. However, it was never shown in FFOCT, neither TD FFOCT or SS FFOCT where full field spatially incoherent illumination and high spectral irradiance are required.

This work will enable to bring spectral shaping to high resolution phase stable OCT configurations. The current configuration described here is particularly dedicated to high resolution imaging, almost 1*μm* in all directions, in particular pushing the boundaries of swept-source OCT systems performances towards high-speed high-resolution systems of promising interest towards volumetric dynamic OCT imaging.

## 2. Vis-NIR FFOCT system design

**Fig. 1.**
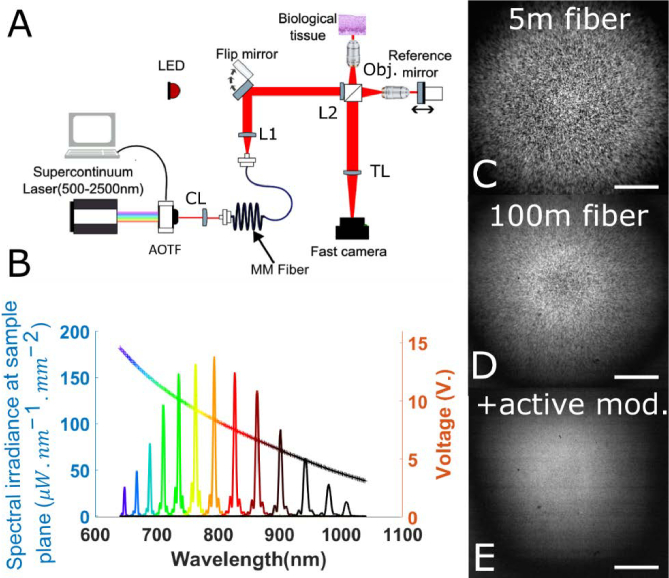
**Tunable wavelength light source for Full Field OCT (FFOCT)**. A. Full Field OCT system layout. A conventional FFOCT system is illuminated either by a standard LED, or by our custom-made spectral shaping module. It is composed of a supercontinuum laser filtered by an AOTF. CL means collimator lens, L1 and L2 are respectively 30 mm and 150 focal length lenses, and TL is a tube lens of 300 mm focal length. Sample and reference arm objectives (obj.) are identical 4X 0.16 NA. B. This illumination module delivers a monochromatic instantaneous spectrum which central wavelength can be tuned at 3μs intervals within the 650-1040 nm range using a voltage analog signal. The monotonously decreasing curve shows the calibration curve linking the voltage to the wavelength (right axis), and the coloured spectra are different instantaneous spectra at a fixed voltage (left axis), expressed as the spectral irradiance at the sample plane. C-E. A multimode mode fiber is used to break the spatial coherence and provide virtual confocal gating in FFOCT, but creates a speckle illumination. Speckle patterns displayed here are the result of a difference between two direct images of the reference mirror taken at different central wavelengths. (C).With a short multimode fiber (2 m) modal interference results in a highly contrasted speckle pattern (D). With a long multimode fiber (100 m), the speckle contrast can be reduced but not completely eliminated (E). A combination of passive (100 m fiber) and active mode mixing results in an homogeneous illumination, only 1.2 times noiser than shot noise measured with the standard LED. Scale bars in (C-E) are 500 μm.

A FFOCT system for high resolution imaging of biological samples was built (Fig. 1(a)). To illuminate the system, we first designed a new specific source module to achieve spatially incoherent illumination with flexible tunable wavelength. It is composed of a supercontinuum laser (Leukos, Electrovis 470) with 10 MHz repetition rate. The laser has an original spectral bandwidth of 470 to 2400 nm, and an average spectral power density around 3 mW/nm in the 600-1000 nm region. It is collimated with a broadband reflective collimator and injected into an acousto-optic tunable filter (Brimrose TEAF5-0.575-1.00-MSD, with a custom-made housing from Leukos) to achieve wavelength tuning. The AOTF has a broad spectral bandwidth from 575 to 1040, but a rather broad filter linewidth (between 2.5 to 9 nanometers (Figure1b)). An RF-driver (Brimrose, VFI-112.5-95-V-A-F2) enables to control the AOTF modulation frequency from an analog voltage input (Fig. 1(c)). Most of this work interest comes from the ability of the RF-driver to achieve a fast-analog wavelength tuning in contrast to most drivers operated with standard RS-232 communication limiting the communication speed to a few milliseconds. This also outperforms the speed of mechanical filters and monochromators by several orders of magnitude. Here, the theoretical AOTF crystal response time is only of 3 µs. Although the RF-driver has a 50 µs constant delay, and the ultrasound propagation time adding another 3 *μs* of constant delay, these delays can be easily compensated for numerically. The light coming out of the AOTF is then injected into a multimode fiber using a pair of silver mirrors (PF05-03-P01, Thorlabs) and an achromatic collimator (A110TM-B, Thorlabs). The multimode fiber is 200µm of diameter and 0.39 numerical aperture (FT200EMT, Thorlabs) resulting in a maximum number of 41 000 spatial modes [31] to produce a spatially incoherent illumination. In order to reduce the speckle pattern contrast at the multimode fiber output (Figs. 1(C)–(E)), a 100 m length fiber is chosen so that each spatial mode propagates with a length difference compared to the next mode (*differential mode delay*) higher than the temporal coherence length [16,31]. Around 50% of the light power is lost during the injection and the propagation in the fiber (with a theoretical loss of 1 dB after 100 meters). While the speckle contrast has been significantly reduced by such a passive mode mixing (Fig. 1(D)) [32], speckle can still be observed, in particular due to the presence of degenerated modes of equal propagation time [33]. To further decrease the speckle contrast, we additionally performed active mode mixing. Approximately 20 meters of the unrolled fiber is actively shaked at around 2 Hz [34] to average several speckle patterns during one exposure time, and achieve a more homogeneous illumination (Fig. 1(E)). Although the frequency of physical modulation is rather low, the frequency of speckle modulation can be increased by increasing the magnitude of the movement, as well as the length of fiber actively shaken. With such combination of passive and active mode mixing, we obtained a spatially and temporally homogenous illumination for exposure times as small as to 2 ms and single wavelength illumination (Fig. 1(E)). We measured a standard deviation of fluctuations only 1.2 times higher than the shot-noise, as measured with the LED illumination. Compared to state of the art SS FFOCT illumination systems [17], we use here a longer camera exposure time and smaller light coherence length (due to the broad filter linewidth of the AOTF), enabling to achieve an almost speckle-free image for both TD FFOCT and SS FFOCT without additional numerical filtering. Altogether, our work displayed in Fig. 1 demonstrates a flexible light source module enabling wavelength tuning over a large range, ranging from visible to infrared illumination with incoherent illumination that can be compatible with FFOCT requirements.

The output of the multimode fiber is collimated by a 30 mm focal length lens (Thorlabs AC254-030-B) and illuminates a conventional FFOCT system [2,3]. Light from the multimode fiber is conjugated with the back-focal plane of objectives by a 150 mm focal length lens placed just before the beam-splitter. The fiber output is magnified by G = 4.17 in both objectives back-focal planes and we obtain a beam of 0.84 mm in the pupil plane. Alternatively, a LED (Thorlabs 850L3) was also used as a light source for comparison. Light sources could be switched by pivoting a flip mirror. Objectives are Olympus 4x 0.16 NA (UPLXAPO) corrected for large spectral bandwidth. A reference silicon mirror is placed at the focal plane of the reference objective. The reference arm is mounted on a motorized translation stage (Zaber T-NA08) and the piezoelectric actuator enables 2 or 4 phases modulation [3]. Samples are placed on a manual XY translation stage and a Z motorized stage (Zaber T-NA08). Tube lens has a focal length of 300 mm giving a magnification of G = 6.67 on a high full well capacity camera (Adimec, Q2HFW) with 1440 × 1400 pixels of 12 µm size each, resulting in a pixel size of 1.8 *μm* and a field of view of 2.5x 2.5 mm^2^ in the sample plane. The spectral irradiance in the sample plan at the brightest wavelength (around 800 nm) is $180\mu W.nm^{-1}.mm^{-2}$ , while the LED spectral irradiance is $62\;\mu W.nm^{-1}.mm^{-2}$ . However, the LED instantaneous spectrum is larger (FWHM of 32 nm) enabling to achieve larger total irradiance than our custom-source module.

**Fig. 2.**
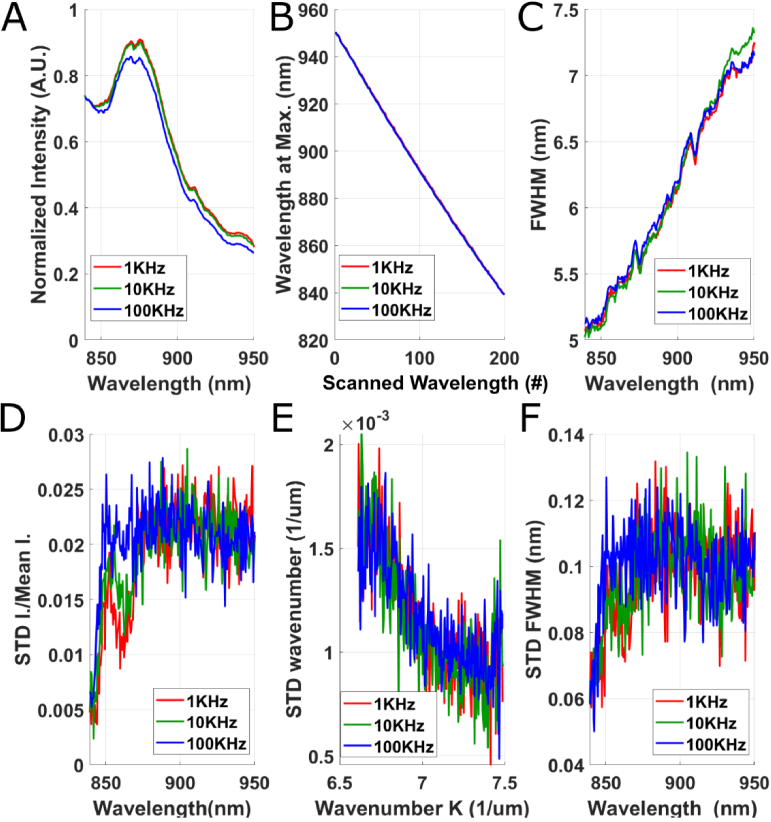
Characterization of the wavelength tuning ability of our module. A-C. Average over 50 measurements of the peak intensity (A), peak wavelength (B), and peak full width half maximum (FWHM- C) while scanning the wavelength with the AOTF. D-F. Standard deviation (STD) over 50 measurements of the peak intensity (D), peak wavelength (E), and peak FWHM (F) while scanning the wavelength with the AOTF.

In order to characterize the ability of the novel source module to perform fast and accurate wavelength tuning, we performed several spectral measurements with a high-resolution holographic spectrometer (HG10, Thorlabs) while sweeping the analog input applied to the AOTF controller at different speeds. The spectrometer exposure time was set at 2*μs*, and was recording the spectrum exiting the source module at respectively, 1, 10 and 100 kHz (Fig. 2). 50 measurements were repeated and the average (Fig. 2 A-C) and standard deviation (Fig. 2 D-F) of respectively the peak intensity, the peak wavelength, and peak full width half maximum (FWHM) for the successive scanned positions are displayed in Fig. 2. We could validate that the wavelength tuning is almost independent from the scanning speed until at least 100 kHz -except from a minor 5% decrease in intensity at 100 kHz (Fig. 2(A)). While the AOTF could theoretically be controlled up to speeds of 300 kHz, we were limited by the spectrometer speed of 100 kHz. We also quantified the fluctuations of intensity and of central wavelength at the output of the source module (Fig. 2(D)–(F)), which are independent from the scanning speed, and correspond to around 2% of intensity fluctuations (Fig. 2(D)), and around 0.1% of error on the instantaneous k-vector (Fig. 2(E)), yielding a phase stability of 21 mrad (as calculated in [35]). This illustrates our ability to perform efficient linear k scanning using the calibration table shown in Fig. 1(B). Finally, we note that while the spectrum in Fig. 2(A) looks different from the spectrum in Fig. 1(B), it is mostly due to the differences of transmission and quantum efficiencies between the spectrometer and FFOCT system.

## 3. Spectral shaping for time domain FFOCT

**Fig. 3.**
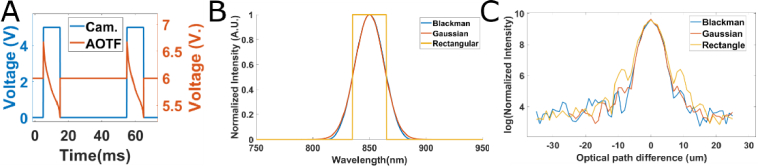
Spectral shaping in TD FFOCT. A. Voltage sequence applied to the AOTF and to the FFOCT camera to perform spectral shaping and obtain a rectangular spectrum. The AOTF command is scanned non-linearly during each exposure time of the camera in order to increase the spectral bandwidth and spend more time on the sides of the spectrum to compensate for the decreasing spectral response. B. Theoretical spectra that we command the custom source to reproduce, showing here rectangular (yellow), Gaussian (red), and Blackman window (blue) shapes. C. Corresponding axial response for the 3 same spectra measured in TD FFOCT by scanning the reference arm length and by looking at interferences with a mirror in the sample arm.

**Fig. 4.**
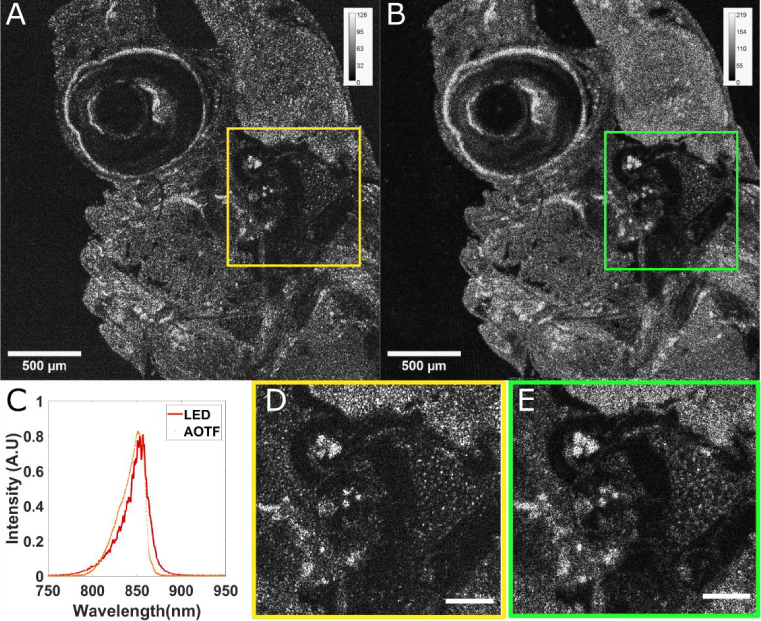
**Spectral shaping for Time domain FFOCT**. A-B. Comparison of time domain FFOCT 4-phases amplitude image of a fixed larval zebrafish at 110μm depth with an LED illumination (A) and our custom source module (B) with identical spectra. C. Experimental spectra of the LED (red) and the AOTF shaped spectrum (orange) as measured with a spectrometer. D-E. Zoom-in regions respectively corresponding to the yellow box in A for the LED illumination (E) and to the green box in B for the custom light source (E) showing a slightly decreased resolution. Scale bars in A and B are 500μm and the total field of view of the zoom-in regions (D-E) is 500 × 500μm^2^.

**Fig. 5.**
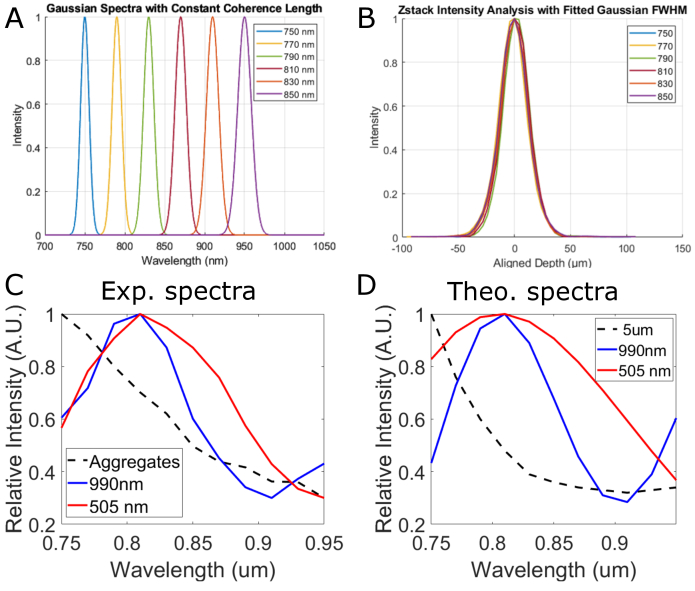
**Spectral shaping enables spectroscopic FFOCT.** A. Simulated spectra to be generated by the spectral shaping module with increasing central wavelengths and spectral bandwidth to keep the temporal coherence length constant. B. Corresponding FFOCT axial profiles obtained with the six spectra of panel A, showing constant coherence length. C. Experimental FFOCT signal of nanoparticles detected from Si0_2_ 990 nm (blue) and 505 nm (red) particles. The median spectrum decreases with wavelength which we postulate to correspond to the presence of numerous aggregates. To extract the contribution of single nanoparticles, we performed a PCA on the measured spectra, and plotted here (blue and red curves) the weighted sum of the three first principal components. D. Mie Simulations of the spectral dependency of the backscattering coefficient of spherical SiO_2_ nanoparticles of diameter 990 nm (Blue) 505 nm (Red), and 5μm to simulate the effect of aggregates.

**Fig. 6.**
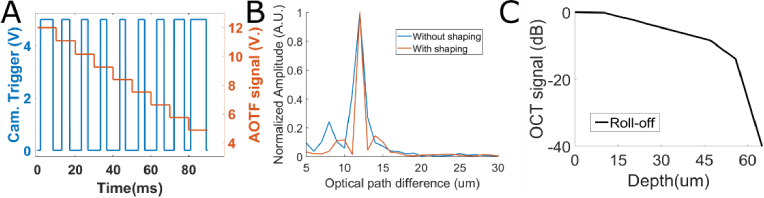
Spectral shaping for SS FFOCT. A. Voltage sequence applied to the AOTF and to the FFOCT camera to perform SS FFOCT and spectral shaping and obtain a rectangular spectrum. The AOTF command (red curve) is swept at increasing constant voltages during each exposure time of the camera, but the exposure time (blue curve- controlled by the duty cycle of the TTL) can be electronically chosen to increase exposure time on the sides of the spectrum and perform spectral shaping. B. Experimental axial responses obtained with SS FFOCT between 650 and 950 nm, without spectral shaping (blue curve) and after flattening of the spectrum (red curve). C. Experimental SS FFOCT signal roll-off, as quantified as the OCT signal amplitude decay between two mirrors for varying optical path differences.

First, we demonstrate the use of our source module to perform spectral shaping for time domain FFOCT (Figs. 3 and 4). In this case, we integrate several wavelengths over one exposure time (Fig. 3(A)) by sweeping the central wavelength exiting the AOTF (Fig. 1(B)) to obtain a broader spectrum (Fig. 3(B)). Thanks to the fast analogic control of the AOTF, we can apply a non-linear function of voltage (Fig. 3(A)) to reproduce any spectrum of interest over one camera exposure time (Fig. 3(B)), and tune the axial response of TD FFOCT (Figs. 3(C), 4(C), 5A-B, and 6B). In Fig. 3(C), we show one example of the effect of such spectral shaping on TD FFOCT axial response. We built 3 consecutive spectra, respectively of rectangular, Gaussian, and Blackman window shapes (Fig. 3(B)), with exactly the same full width half maximum (FWHM). We then performed a scan of the reference arm length in TD FFOCT while imaging a mirror in the sample arm. Figure 3(C) shows the axial response in log scale with the 3 spectra, showing both the ability to achieve the same axial resolution, but with reduced side lobes by almost 10 dB from the rectangular spectrum (yellow curves) to the Gaussian spectrum (red curves) and by another 10 dB with the Blackman window (blue curves).

To demonstrate the effect of spectral shaping and to show that our source module with lowered spatial coherence can achieve high quality TD FFOCT in a real biological sample, we imaged the same fixed larval zebrafish with a conventional high power 850 nm LED (Fig. 4(A)) and with the custom light source (Fig. 4(B)) tuned to send the identical spectra. Hence, we aim here to compare both images only in terms of spatial resolution, cross-talks and other speckle-related effects. To reproduce the LED spectrum, we applied each voltage (and its corresponding wavelength) for a fraction of the camera exposure time proportional to the LED normalized power spectral density. Using a spectrometer (Thorlabs CSS175/M), we showed that we successfully reproduce the same spectrum as the LED with the custom source (Fig. 4(C)), illustrating further our ability to reproduce any spectrum shape. The small spectrum errors are due to the fact that we did not consider the instantaneous AOTF linewidth to reconstruct the final spectrum, which could be easily implemented in future work.

Figure 4 highlights the ability of our source module to achieve high quality time domain FFOCT images. Figures 4(A) and 4(B) compare time domain 4 phases FFOCT images (with 50 accumulations) at 110 µm depth inside a fixed zebrafish larva under a glass coverslip with illumination respectively from the LED and from the supercontinuum swept source module. Zoom-ins corresponding to the same central region are shown in Figs. 4(D) and 4(E). Qualitatively, both images show comparable contrast and image quality demonstrating the ability of our source module to significantly reduce speckle and spatial coherence. More quantitatively, we measure a tomographic signal between 1.5 and 2 times higher when the laser source is used compared to the LED illumination.

We also observe a slightly decreased transverse resolution when the laser source is used. By taking into account the refractive index mismatch between the air objectives and the sample, resulting in significant optical aberrations at 110*μm* depth, together with the higher degree of spatial coherence ( $4.10^{4}$ modes, and NA of 0.39 vs. around $10^{6}$ for the LED [36] and 90° of viewing angle- corresponding to a fully spatially incoherent illumination) these observations are consistent with the theory on aberrations in FFOCT [15]. Besides, the laser beam is only covering about 1 mm of the pupil plane of the objective, which is not critical for the sample arm since the scattered light covers the full objective pupil, but even without aberrations, a $\sqrt{2}$ -fold reduction of transverse resolution is expected [15]. This could be fixed by adjusting the magnification between the fiber output and the pupil plane. We finally observed that both the signal increase and the resolution loss become stronger with increasing depth (Not shown here) using the custom source versus the LED, illustrating the effect of increasing aberrations and the effect of pupil size and spatial coherence.

## 4. Towards quantitative spectroscopic FFOCT

Using the spectral shaping enabled by our source module, we performed a proof-of-principle experiment of spectroscopic FFOCT [37] (Fig. 5). By changing the applied voltage sequence to the AOTF, both the central wavelength and the spectrum width can be tuned independently. This allows studying how FFOCT signal varies with the wavelength, while maintaining a constant temporal coherence length $\left(l_{c}∼\frac{\lambda_{0}^{2}}{\Delta \lambda }\right)$ , to keep the same region imaged. This is of critical importance because subtle spectral responses could be missed due to the increase of the coherence volume generally observed in commercial LEDs as the central wavelength goes deeper in the infrared. We created several spectra of fixed coherence length with increasing central wavelengths from 750 to 950 nm (Fig. 5(A)). The corresponding FFOCT axial profiles measured on a mirror are all superimposed (Fig. 5(B)) illustrating the constant temporal coherence length, and again the interest of our spectral shaping strategy.

To illustrate the interest of our such spectroscopic FFOCT measurements, we imaged monodispersed 990 nm and 505 nm diameter polystyrene nanoparticles (PolyBead MicroSpheres # 07310-15, Polysciences, USA) dispersed in an agar gel $\left(C=3.10^{-6}g/mL\right)$ (Fig. 5(C)) with the microscope described in Fig. 1. According to Mie simulations we performed using Mie Python software [38], the backscattering coefficient of these particles (proportional to the FFOCT signal) display significantly different spectral variations for different radii (Fig. 5(D)- blue vs red curves). Experimentally, we measured FFOCT signal of the nanoparticle phantom at 11 different central wavelengths (from 750 to 950 nm every 25 nm with a constant coherence length of 10.6 *μm*).

We achieve the same level of global saturation of the camera with a maximum deviation of 2% for each acquisition thus allowing to neglect the wavelength dependency of the camera’s quantum efficiency and the spectral variations of reference mirror reflectivity. Tomographic images were acquired with four phase demodulation and we used the amplitude signal for the following analysis. Particles were automatically segmented using iLastik software [39] and analysed with Matlab. For 990 nm beads, 145 particles could be detected and we obtained 145 spectra of 11 spectral points, with a diffraction-limited resolution (average measured diameter of 3.0*μm* at 750 nm, linearly increasing with wavelength). The maximum intensity value within each segmented region is extracted and plotted versus wavelength. Unfortunately, we could not prevent the formation of aggregates of nanoparticles, which result in a median spectrum significantly different from theory (Fig. 5(C) -black dashed line for the experimental curve vs. Figure 5(D) red and blue curves for the expected theoretical responses). Going back to theoretical simulation, the spectral response of larger SiO_2_ particles (5*μm*) look similar to the experimental curve we obtained (See black dashed line in Fig. 5(D) for simulation, resembling the experimental curve in Fig. 5(C)). To extract the contribution of single nanoparticles in the experiments, we performed a PCA (using the dedicated *pca* function from Matlab, *Mathworks*) on the matrix 145 × 11 containing the 144 particle spectra and calculated the weighted sum of the three first principal components respectively corresponding to 47%, 17%, and 15% of the total energy for the 990 nm beads. With such filtering strategy, we obtain experimental spectra (Fig. 5(C) blue and red curves) corresponding to the theoretical predictions. Hence, we achieved spectroscopic measurements enabling to differentiate single sub-diffraction limit nanoparticles of different sizes and to add a new contrast to FFOCT images. Indeed, the non-linearity of Mie scattering with particle size in such size regime would prevent the use of intensity-based segmentation of nanoparticles.

## 5. Wavelength-tuning and shaping for swept source FFOCT

Alternatively, we demonstrate that the developed source module can be used to achieve high speed SS FFOCT at central wavelengths and spectral bandwidths that are not found in commercial swept source lasers. For SS FFOCT, we scanned a maximum bandwidth of [650; 950] nm in 250 successive 1440 × 1440 images acquired in 1 second. The relatively low frame-rate of 100 fps is here limited by the camera we had available on the current setup, but the wavelength scanning could be achieved up to about 100 kHz, as shown in Fig. 2. Each image was recorded at a different central wavelength with minimal filter linewidth (see Fig. 1(B)), scanned linearly in k by changing sequentially the AOTF input voltage for each image (Fig. 6(A)). In order to reduce the spectrum variation, and increase axial resolution, spectral shaping can also be achieved in SS FFOCT with our source module. In this configuration, the spectrum cannot be directly shaped since only one wavelength is sent per exposure time, but a varying camera exposure time can be implemented by changing the duty cycle of TTL trigger signal of the camera (Fig. 6(A)), enabling to overexpose the sides of the spectrum and equalize the spectrum over the full bandwidth [650 nm; 950 nm]. This results in a theoretical axial resolution (full width half maximum of a cardinal sinus) $Res_{Z,\;theo}≃0.6\frac{\lambda_{0}^{2}}{\Delta \lambda }≃1.2\;\mu m$ .

Experimentally, after spectral zero-padding to increase axial sampling, we measured on a mirror at 12 *μm* of optical path an axial resolution of 1.5 *μm* without the spectral shaping and 1.1*μm* after spectral flattening, demonstrating the interest of spectral shaping for SS FFOCT (Fig. 6(B)). However, the price to pay for this enhanced axial resolution and associated large bandwidth is the limited penetration depth. First, to reduce acquisition time, we recorded only 250 images, enabling to reach a maximum depth range of 65 *μm*. Second, the large filter linewidth (between 4 and 9 nm from 650 to 950 nm) results in a single wavelength coherence length of 54 to 72 µm and creates a strong roll-off experimentally measured at -13.9 dB at 55 µm (Fig. 6(C)).

Finally, we aim to demonstrate that our source module can efficiently acquire volumes in SS FFOCT in biological samples. After sample positioning, we acquired 250 different wavelengths linearly spaced in k, using the precise wavelength calibration shown in Figs. 1 and 2. Prior to any measurement, we also record the corresponding reference volume by blocking the object arm, and subtract the two stacks to remove most of the incoherent light and avoid saturation of the zero-th order of the spectral Fourier transform. We multiplied the interference spectra by a Hamming window and perform a direct Fourier transform in the k dimension to obtain the 3D complex amplitude [8,9]. Surface of the sample was placed near the zero optical path difference to avoid overlapping of the complex conjugate.

**Fig. 7.**
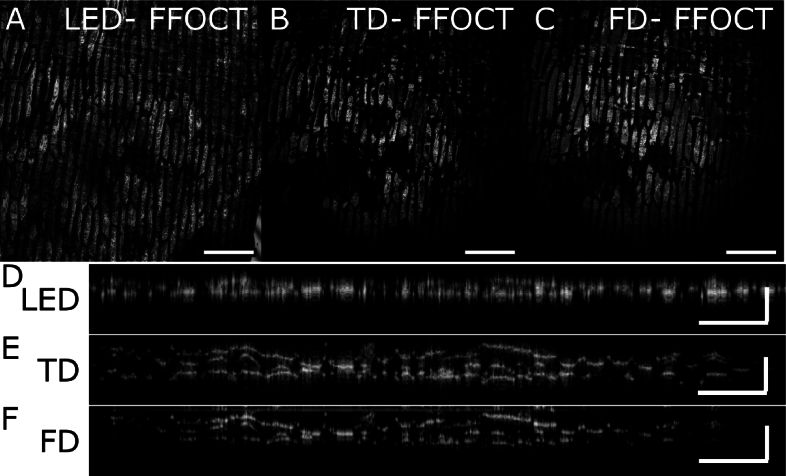
**SS FFOCT performances for biological imaging. A-C.** Comparison of En face image of an onion skin at 40μm depth for LED TD FFOCT (LED- A) custom-source TDFFOCT( TD- B) and SS FFOCT (FD - C) (C) D-F. Corresponding axial views of the same sample on a line at the centre of the field of view. FFOCT performed with our custom source spans from 650 to 950 nm, with 250 images spaced linearly in k-space, resulting in a measured axial resolution around 1 µm. All horizontal scale bars correspond to 250 μm and vertical bars in D-F to 40 μm.

Figure 7 shows *en face* views and B-scans of onion skin, imaged with both TD FFOCT with LED illumination aiming to illustrate the low spatial coherence case, TD FFOCT with our custom source (Figs. 7(B), 7(E)),tuned to illuminate the full [650;950]nm bandwidth, and with SS FFOCT (Figs. 7(C), 7(F)). FFOCT with our custom source module shows 5-times higher axial resolution as the current LED used, enabling to clearly detect cell boundaries in 3D. To compare the *en face* views, we averaged 7 planes in TD and SS FFOCT to obtain similar axial range as LED-based FFOCT. Qualitatively, *En face* images measured at 40 µm depth show similar contrast and resolution in all modalities showing little to none cross-talk and wavelength-dependent speckle effects, and the capacity of our multimode fiber and mode mixing to achieve high quality SS FFOCT at such microscopic resolution for the first time.

## 6. Discussion and conclusion

We developed a new source module for FFOCT combining a supercontinuum laser and an AOTF with a very large bandwidth and a fast tuning speed. The obtained central wavelength and bandwidths of the light source have no equivalent commercial swept-source solutions enabling exploring historically challenging new regimes of FFOCT imaging, such as spectroscopic OCT [37], or visible-light swept-source OCT [40]. SS FF OCT was also demonstrated at an unprecedented 1.0 µm axial resolution. In order to achieve high image quality, we injecting the light in a long multimode fiber with 40,000 spatial modes and use passive and axial mode mixing to successfully remove the effects of spatial coherence and wavelength-dependent speckle variations. Also, in terms of FFOCT instrumentation, we reported on the necessity to use optical elements corrected for extended spectral bandwidth, in particular for the microscope objectives to reduce chromatic aberrations on such large 400 nm large bandwidth.

Compared to other swept source OCT instruments, our system enables ultra-high resolution in all 3 dimensions, and to perform subcellular resolution imaging. The drawback of the current design is the limited imaging range around 60*μm*, which would prevent its use for several OCT applications such as *in vivo* retinal imaging for instance. The combination of such device with a longer depth range system, such as point scanning low resolution OCT system as in Mecê et al. [41] to track and compensate for retinal movements in real time could however solve the issue. We also note that the limited depth range primarily depends of the choice of the AOTF, and by the instantaneous linewidth. AOTFs with shorter linewidth could be chosen at the cost of an even more reduced power. To mitigate this point, most commercial swept source lasers use semiconductor optical amplifiers (SOA), which however limits the spectral bandwidth. As a possible future workaround, sources with higher spectral irradiance could be used, such as laser-driven light sources, together with AOTFs showing smaller linewidth.

For TD FFOCT, our module is (much) more complex and expensive compared to traditional LED or thermal sources typically used, while not necessarily achieving higher axial resolution. Its spectral irradiance is higher, but since most LEDs have large spectral bandwidth, the total surface power density is typically higher with LEDs. However, our current configuration could more easily be scaled-up in terms of power, for instance by choosing higher power supercontinuum or brighter sources such as laser driven plasma sources. Surpassing the illumination power compared to traditional light sources would be of significant interest, since it has now become the current bottleneck of TD FFOCT. Alternatively, more flexible but slower and more complex spectral shaping solution for TD OCT, for instance using a grating coupled to a DMD to have the ability to transmit several wavelengths at once [30] could be implemented and combined with methods to break the spatial coherence in order to benefit from the larger spectral irradiance.

Nonetheless, we believe that the main advantage of our source module is its ability to perform spectral shaping on large bandwidths, unlocking the possibility to perform quantitative spectroscopic FFOCT measurements, without affecting the size of the measured volume.

For SS FFOCT, our custom module source also has advantages and drawbacks. On the one end, we can benefit from a large spectral bandwidth, but this at the cost of the absence of narrowband optical amplifiers (typically yielding power 10 times lower than commercial swept source lasers), and of a slightly increased error on the instantaneous wavelength (typically by a factor 3), but still keeping a relatively good phase stability. Shall higher phase stability be required, it would be possible to improve it either by coupling the current system with a second point-scanning interferometer to measure the instantaneous phase as in [42], or to use some optical reflections to perform a simultaneous measurement of the peak wavelength, and hence to correct for their variations. We also believe that most of the spectral fluctuations we have are due to the electrical noise (around the dozen of mV) of the analog input signal, which could be decreased with more advanced signal generators or with adequate filters.

The main current drawback of our module is its more limited depth range, smaller by a factor 70 compared to commercial sources, due to the large filter linewidth, limiting the applications of our current configuration to microscopy and biological applications, such as explants and *in vitro* systems.

Regarding the sweep speed, our custom module can have sweep speeds ranging from <1 nm/s to 10^6^ nm/s, slightly exceeding commercial swept sources. Yet, we believe that the main advantage of our module lies in its ability to scan large spectral bandwidth for high resolution SS FFOCT. First, thanks to the increased spectral bandwidth by a factor compared to commercial alternatives, the axial resolution is also increased by the same factor allowing what we believe to be the highest axial resolution ever achieved in swept source OCT (and a fortiori in swept source FFOCT). Besides, no commercial swept-source laser is available in the visible domain, while it would be possible with our source (and even more if visible AOTF were used to replace the one in our module), enabling to enhance scattering and to increase resolution. Finally, our module offers a flexibility to perform both linear and non linear k scanning, and to perform spectral shaping, which is, to the best of our knowledge, not possible with commercial swept-source lasers.

To summarize, with such a novel source module, it opens a number of new applications for biomedical imaging, mostly restricted to high resolution imaging due to the penetration depth constraint. In time domain FFOCT, it enables to increase the illumination power compared to traditional light sources, which has become the current bottleneck, without sacrificing the temporal or spatial coherence. Besides, it permits to adapt the illumination spectrum to each sample for optimal performances, and to provide spectroscopic information. Finally, thanks to the fast spectrum scanning, up to 100 kHz and to the large bandwidth of the AOTF, high speed swept source FFOCT becomes accessible at high axial resolution. This is a key advancement towards volumetric dynamic FFOCT in real time, itself a key milestone towards in vivo dynamic OCT.

## Data Availability

Data underlying the results presented in this paper are not publicly available at this time but may be obtained from the authors upon reasonable request.
